# In-situ atomic level observation of the strain response of graphene lattice

**DOI:** 10.1038/s41598-023-29128-4

**Published:** 2023-02-11

**Authors:** Jz-Yuan Juo, Bong Gyu Shin, Wolfgang Stiepany, Marko Memmler, Klaus Kern, Soon Jung Jung

**Affiliations:** 1 Max-Planck-Institut für Festkörperforschung, Heisenbergstraße 1, 70569 Stuttgart, Germany; 2 SKKU Advanced Institute of Nanotechnology (SAINT), Sungkyunkwan University (SKKU), Suwon, 440-746 Republic of Korea; 3 Institut de Physique, École Poly-technique Fédérale de Lausanne, 1015 Lausanne, Switzerland

**Keywords:** Mechanical and structural properties and devices, Scanning probe microscopy

## Abstract

Strain is inevitable in two-dimensional (2D) materials, regardless of whether the film is suspended or supported. However, the direct measurement of strain response at the atomic scale is challenging due to the difficulties of maintaining both flexibility and mechanical stability at low temperature under UHV conditions. In this work, we have implemented a compact nanoindentation system with a size of $\sim$ 160 mm$^2$ $\times$ 5.2 mm in a scanning tunneling microscope (STM) sample holder, which enables the reversible control of strain and gate electric field. A combination of gearbox and piezoelectric actuator allowed us to modulate the depth of the indentation continuously with nanometer precision. The 2D materials were transferred onto the polyimide film. Pd clamp was used to enhance the strain transfer from the polyimide from to the 2D layers. Using this unique technique, strain response of graphene lattice were observed at atomic precision. In the relaxed graphene, strain is induced mainly by local curvature. However, in the strained graphene with tented structure, the lattice parameters become more sensitive to the indentor height change and stretching strain is increased additionally. Moreover, the gate controllability is confirmed by measuring the dependence of the STM tip height on gate voltage.

## Introduction

Research into two-dimensional (2D) materials has already revealed surprising results often disregarded in the third dimension. Instead of a simple planar surface, the 2D materials have turned out to contain a rich landscape of corrugations when suspended^1,2^. When placed on a substrate, the strain is induced by the lattice mismatch^3^ or simply from the surface morphology^4,5^. For MoS$_2$, the strain can modulate the band gap^4,6^, spin-orbit coupling^7,8^, thermal conductivity^9,10^, effective masses of charge carriers^11^, and energy barriers for structural phases^12,13^. Moreover, strain can induce giant pseudomagnetic fields in graphene^14,15^, and can optimize the catalytic efficiency of transition-metal dichalcogenides for hydrogen evolution reactions^16,17^. All of these properties are critical for the use of 2D materials in future applications such as mountable physiological monitoring devices^18,19^, touch screens^20,21^, flexible energy storage systems^22–24^, catalysis^25–28^, and batteries^29–31^. Since the strain has variation at the nanoscale^4^, the direct observation of the electronic structure at the atomic scale is required to characterize the impact of strain on individual atoms. Therefore, a wide range of strategies has been employed for applying strain to 2D materials for scanning tunneling microscopy (STM) measurements, including the use of pre-patterned structures^32–34^, piezoelectric-based devices^35,36^, and dual-probe setup^37^. However, most of the reported devices suffer limited strain range or poor stability.

**Figure 1:**
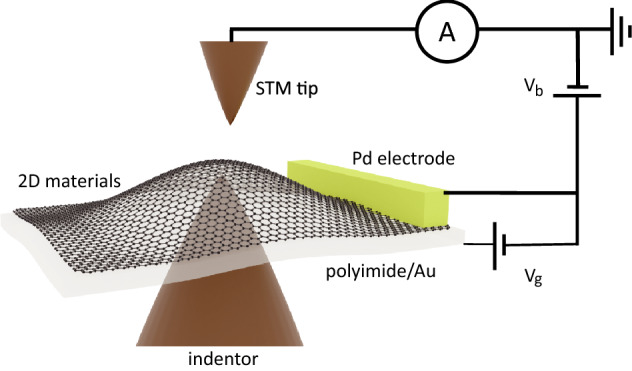
The schematic of strain- and gate-controllable STM structure. V$_b$ and V$_g$ are bias and gate voltages applied on the sample, respectively.

In this work, a new STM sample holder has been developed for subjecting 2D crystals to controllable strain and gating. Its working principle is based on the nanoindentation method^2,38^ (Fig. 1). 2D materials including monolayer MoS$_2$ and graphene were transferred using the wet etching method^39^ onto a flexible polyimide film and fixed, and then controllably deformed by the application of an indentor. The combination of gearbox and piezoelectric actuator allowed us to control the distance between indentor and sample continuously with nanometer precision. The polyimide film with a thickness of $\sim$ 1 $\upmu$m also served as the dielectric to enable the electrical gating of the 2D materials. To form a gate structure, a thin Au layer was deposited at the bottom of the polyimide film as a back contact and a Pd electrode was deposited on top of the 2D material.

Our method exploited the advantages and simplicity of well-established nanoindentation methods, which have been successfully applied in a number of previous studies on 2D materials^2,37,38,40,41^. The advantages of our method include: (a) Using a piezoelectric actuator, the indentor height can be controlled with a precision of <1 nm. (b) The gearbox has a travel range of $\sim$ 120 $\upmu$m, increasing the dynamic range of the applied strain. (c) Since the 2D materials are supported by the polyimide film and indentor, their vibration is negligible, thus allowing atomic resolution measurement. (d) All the materials used are compatible with UHV and cryogenic environments. (e) The compact holder, with a size of $\sim$ 160 mm$^2$ $\times$ 5.2 mm, is compatible with a wide range of measurement techniques. For example, it can be used in atomic force microscopy (AFM), which enabled us to measure the macroscopic deformation of the 2D materials. Moreover, by monitoring the shifts of the Raman peaks, the strain transfer efficiency was quantified. In this way, we can relate macroscopic measurement results to the atomic scale STM measurement with controllable strain. Using this technique, we examined the strain response of the graphene lattice with atomic precision. The deflection of the graphene was traced by the variation of STM tip height in the constant current mode. The dependence of the deformation of the graphene on the indentation depth was quantified. Finally, we analyzed the strain response of the graphene lattice parameters from the series of atomic resolution STM images taken during strain modulation. This direct observation of graphene lattice changes caused by strain provides valuable information for developing straintronics in 2D materials.

## Results and discussion

### Sample holder design

**Figure 2:**
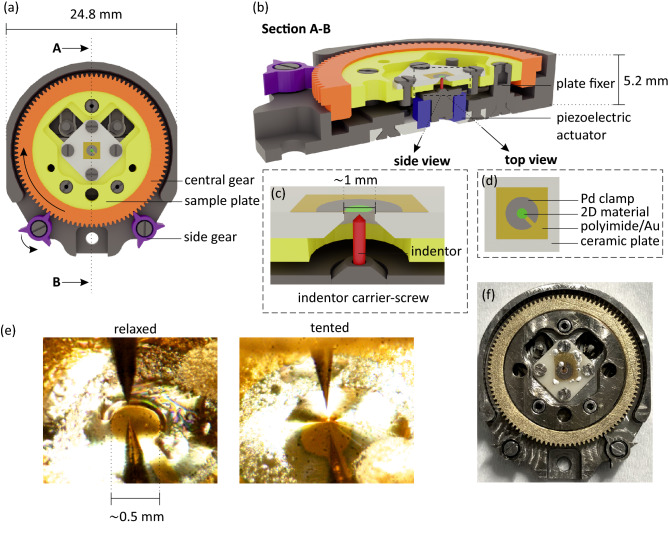
Design of strain- and gate-controllable STM sample holder. (**a**) 3D model top view. (**b**) Cross section of A-B line drawn in (**a**). (**c**) Detailed side view of the strained junction. (**d**) Top view of the sample area. (**e**) Optical images of STM tunneling junction where the strain conditions of sample are controlled using gearbox system. (**f**) Photograph of the sample holder.

Figure 2 shows the schematic overview of our strain- and gate-controllable STM sample holder. The basic principle of the strain application follows. An electrochemically etched W tip is mechanically ground with an apex radius of a few $\upmu$m (red in Fig. 2b, c) and used as an indentor to exert force on the 2D material (green in Fig. 2a–d). The 2D material is first transferred onto a polyimide film with a thickness of $\sim$ 1 $\upmu$m. Then, the 2D material/polyimide film is transferred and then glued to the ceramic sample plate with a central $\sim$ 1 mm diameter circular aperture (Fig. 2c, d). The strain is applied by controlling the relative positions of sample and indentor.

We use a gearbox in combination with a piezoelectric actuator to obtain a large travel distance with high precision. A gearbox is positioned (orange and purple in Fig. 2a, b) inside the sample holder body (grey). The gearbox design is based on a “differential adjusting” concept. The central gear (orange in Fig. 2a, b) has 100 teeth, and its thread pitch at the outer and inner sides are 0.30 and 0.25 mm/revolution, respectively. As the sample plate (yellow in Fig. 2a–c) is not rotatable, the central gear moves unequally in the opposite direction to the sample plate. Therefore, the sample height, which depends on the difference between inner and outer thread pitch of the central gear, can be precisely controlled. Two side gears (purple in Fig. 2a, b), with three separated teeth that partly protrude outside the sample holder body, are designed for the in-situ rotation using the wobble stick inside the UHV chamber. Each separated tooth of the side gears enables the precise angular control of the central gear tooth-by-tooth. As a result, the height of the sample plate can be positioned with a travel range of $\sim$120 $\upmu$m and a precision of $\sim$ 1.4 $\upmu$m. Additionally, a circular piezoelectric actuator (blue in Fig. 2b) is integrated and provides a travel range of $\sim$ 1.8 $\upmu$m with < 1 nm precision at room temperature.

The keyhole-like Pd clamp with an inner diameter of $\sim$ 0.5 mm and a thickness of $\sim$ 30 nm deposited on top of the 2D material enables efficient strain transfer from the polyimide film^42^. To form a gate structure, a $\sim$ 20 nm Au layer is deposited at the bottom of the polyimide film as a back contact. Figure 2e shows the optical images of relaxed graphene/polyimide film with relatively low strain and one with a teepee tent-like shape (referred to as “tented” below) in high strain, applied using the gearbox based control system. To go from the relaxed structure to the tent shape, the central gear is rotated by one tooth of a side gear $\sim$ 5 times. The photograph of the assembled sample holder is shown in Fig. 2f.

### Macroscopic scale measurement

**Figure 3:**
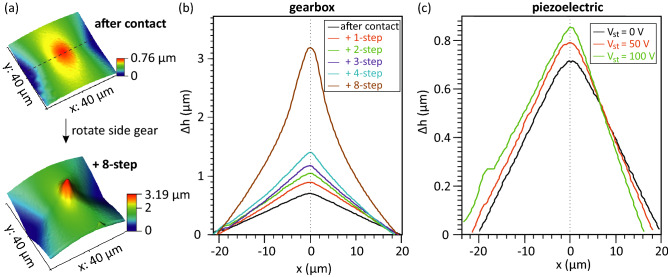
Height controllability of the sample and the indentor using gearbox and piezoelectric actuator (**a**) AFM topography images of polyimide surfaces taken before and after moving the polyimide film down by 8-steps using the gearbox. A step is defined as the height change when one tooth of a side gear is moved. (**b**) Height changes by gearbox control. Line profiles are taken from the center of the deformed area, shown as the black dotted line in (**a**). $\Delta$h is the height difference between the highest and lowest points in the image. The position with the maximum height is defined as the center point ($x=$ 0). (**c**) Height changes by piezoelectric control. The piezoelectric actuator moves around 1.8 $\upmu$m under an applied voltage (V$_{st}$) of 100 V at room temperature.

As shown in Fig. 2, the height of the sample plate can be controlled by the gearbox, and the height of the indentor can be controlled by the piezoelectric actuator. The strain can be increased by either lowering the sample plate or raising the indentor. AFM results in Fig. 3 show the height controllability of the sample plate and indentor in the sample holder by using the gearbox and piezoelectric actuator, respectively. Figure 3a, b shows how the polyimide film before transferring the 2D material is deformed by moving the sample plate down using the gearbox. Due to the limited lateral range of the AFM (max $\sim$ 40 $\times$ 40 $\upmu$m$^2$), the entire deformed polyimide film inside the circularly clamped area ($\sim$1 mm in diameter) cannot be imaged in a single AFM image. Therefore, we use the relative height change ($\Delta$h), the difference between the highest and lowest points in the image, to compare the shape of the deformed surfaces with a range of sample and indentor heights. To do this, the sample plate is moved downwards in a step-by-step manner using the gearbox-based control system, and the height change, $\Delta$h, is monitored. With increasing gear steps from +0 (after contact) to +4, $\Delta$h increases from 0.77 to 0.93, 1.06, 1.19, and 1.40 $\upmu$m. A tent-like deformation is shown in the topographies and line profiles^43^. When the applied strain is increased further by increasing the number of gear steps to +8, the relative change in $\Delta$h increases dramatically, indicating that the deformation of polyimide film is more sensitive to the sample plate height under conditions of higher strain. Moreover, the topography changes from a tent-like structure to a bubble-like structure at an applied strain equivalent to +8 gear steps^43^. Figure 3c shows the dependence of $\Delta$h on the position of the indentor under the control of the piezoelectric actuator. The indentor height can be controlled with nanometer precision via the applied voltage to the actuator (V$_{st}$). As the maximum travel range of the piezoelectric actuator ($\sim$ 1.8 $\upmu$m) is larger than the gearbox precision ($\sim$ 1.4 $\upmu$m), the indentor-sample distance can be tuned continuously over a range of $\sim$ 120 $\upmu$m while maintaining nanometer precision.

**Figure 4:**
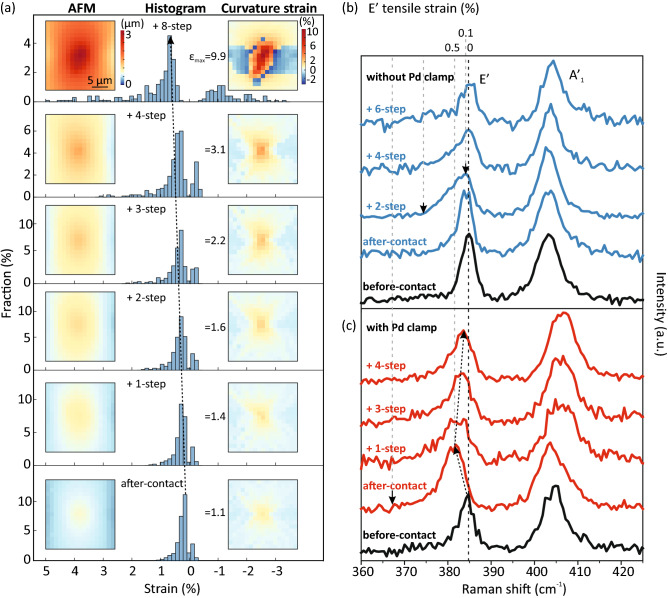
Characterization of strain distribution. (**a**) Calculation of the curvature-induced strain distribution based on the AFM topography images measured on the polyimide film before transferring the 2D material that were shown in Fig. 3. (**b**, **c**) Raman spectra taken at the indented center of monolayer MoS$_2$ transferred on polyimide film with increasing the number of gear steps. (**b**) is with and (**c**) is without a Pd clamp.

To verify how much strain can be applied and its distribution in the indentation process, we first analyze the curvature-induced strain distribution on the bare polyimide film before transferring the 2D material (Fig. 3), as shown in Fig. 4a. As the number of gear steps are increased, the maximum strain increases gradually from 1.1% (after-contact) to 9.9% (+ 8-step), and the strained area becomes broader. Indentation-induced deformation is localized and heterogeneous. The region on top of the indentor is under tensile stress and is surrounded by the area under compressive stress. The curvature-induced strain approaches zero with increasing distance from the contact region.

It is reported that the maximum strain ($\varepsilon _{max}$) that can be applied on 2D materials through the supported polymer substrate is only 0.6-3.2%. Beyond this critical strain, 2D layers begin to slip and decouple from the substrate^42,44–51^. Therefore, to verify how much strain can be transferred from the polyimide film to the 2D material in our indentation system, we conduct the Raman spectroscopy measurement. To identify the peak shifts due to strain, monolayer MoS_2_ is used as the test 2D material here due to its stronger optical contrast and Raman signals than, for example, graphene, on the polyimide film. As the sample surface is not flat in the process of indentation, we use a 10 × objective, which has a longer depth of focus, to focus a 488 nm laser beam on the indented center with a 1/e^2^ spot size of $\sim$ 10 $\upmu$m to maximize the Raman signals.

Figure 4b shows the Raman spectra of monolayer MoS$_2$/polyimide film. Before engaging the indentor, the monolayer MoS$_2$ shows the E$^\prime$ mode peak located at 384.8 cm$^{-1}$ and the A$^\prime$ mode peak located at 403.2 cm$^{-1}$. Since the peak position of the E$^\prime$ mode is more sensitive to the strain applied through bending (5.2 cm$^{-1}$/%) than the A$^\prime$ mode (1.7 cm$^{-1}$/%)^52^, we use the shift of the E$^\prime$ mode to characterize the curvature-induced strain ($\varepsilon$) transferred from the polyimide film to monolayer MoS$_2$ and compare this with the indentation depth. Taking the peak position before contact as our zero-strain reference, the E$^\prime$ peak shows a redshift of $\sim$ 0.6 cm$^{-1}$ when the gearbox is rotated + 2-step, which corresponds to $\varepsilon \sim$ 0.1%. It is worth noting that the E$^\prime$ has an asymmetric distribution with the tail of the peak extending to $\sim$ 375 cm$^{-1}$, and its asymmetry increases with increasing gear steps. However, as the observed asymmetry of the E$^\prime$ peak can be induced together by the non-uniform strain distribution and the splitting of peaks^45^, it is challenging to define the maximum strain from the peak fitting or its tail position. When the gearbox is rotated $\ge$ 4 steps, the E$^\prime$ peak returns toward the reference position, indicating the strain relaxation possibly due to the formation of wrinkles or cracks^53^.

To enhance the strain transfer, the MoS$_2$ monolayer is clamped onto the polyimide film by evaporating Pd layer on the edge of MoS$_2$ monolayer through a circular-shaped shadow mask^42^. Figure 4c shows the Raman spectra of monolayer MoS$_2$ with an additional circular Pd clamp deposited on top. By virtue of the additional clamping, the E$^\prime$ peak in the after-contact case shows a larger redshift of $\sim$ 3 cm$^{-1}$, corresponding to $\varepsilon \sim$ 0.5%, and the peak tail extends further to $\sim$ 368 cm$^{-1}$, which confirms that the Pd clamp enables more efficient strain transfer from polyimide film to monolayer MoS$_2$. The shift of the E$^\prime$ peak position back to the reference position with increasing gear steps is again noted, showing a similar behavior of gradual relaxation.

In short summary, as the indentation depth increases, the curvature-induced strain increases inhomogeneously, observed in both the AFM and Raman results. The strain transfer can be enhanced by the addition of a Pd top clamp.

### Atomic scale characterization

**Figure 5:**
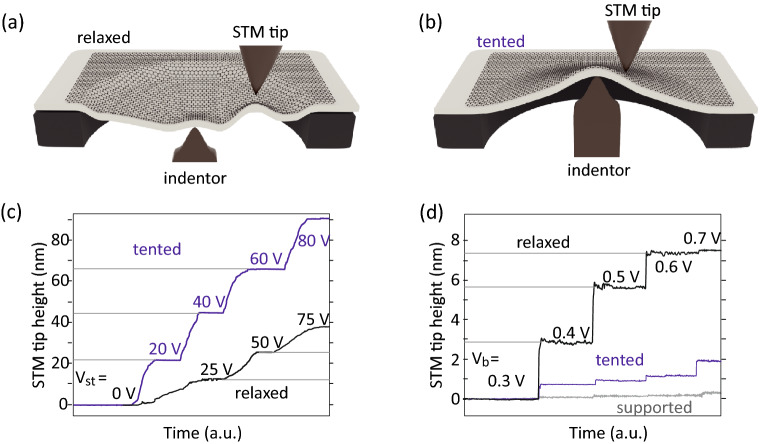
STM measurement on the graphene transferred on polyimide film. (**a**) Strain-free (relaxed) condition and (**b**) tented distortion induced by strain applied using the gearbox based control system. The sample height dependence on the piezoelectric actuator voltage (V$_{st}$), and the sample bias voltage (V$_b$) are shown in (**c**) and (**d**), respectively.

Using the strain-controllable sample holder, the indentation response of the graphene is studied with UHV STM at $\sim$4.8 K. Although the indentation depth can be controlled with nanometer precision, this cannot be directly translated to the applied strain on 2D materials during STM measurement. Figure 5a, b shows the schematics of graphene transferred onto polyimide film under relaxed and strained conditions during STM measurements. After contact and before gearbox rotation, i.e. in the relaxed case, the strain is localized on top of the indentor, and the remaining area of the film lies below the aperture (Figs. 2e, 5a). In the high strain case after the gearbox rotation, the indentor exerts force on the whole polyimide film, and a tent-like structure is formed (Fig. 2e, 5b).

At such high strain, the film is tightened and becomes more sensitive to the indentor height. The deformation of the 2D material depending on V$_{st}$ is characterized by measuring the z movement of the STM tip in the constant current mode. When the indentor pushes up the graphene/polyimide film, the STM tip retracts accordingly to maintain the current constant, as shown in Fig. 5c. For the relaxed condition, the STM-tip retracts (37 nm) when V$_{st}$ increases from 0 to 75 V. However, in the tented case, a larger V$_{st}$-dependent sample height change (85 nm) is observed when V$_{st}$ increases from 0 V to 80 V. The sample height change dependence on V$_{st}$ can be controlled reversibly (Fig. S1). The absence of hysteresis in the height change suggests that the polyimide film is still flexible even at $\sim$ 4.8 K, and the slippage between indentor and polyimide film is negligible.

On the other hand, the relaxed graphene/polyimide film is easily affected by external stimuli in comparison with the tented case. Figure 5d shows the change in STM-tip height with increasing V$_b$ from 0.3 to 0.7 V. The recorded heights are 0.2, 1.8, and 7.3 nm for the relaxed, tented, and supported cases, respectively. In comparison with the supported area where the STM-tip height is determined solely by the electrostatic force, a more considerable STM-tip height change in the suspended area is induced by the additional height change of the graphene/polyimide film pulled up by the STM-tip (via the van der Waals forces)^54^. A much larger height change is observed in the relaxed case, since the polyimide film has a lower tension, as shown in Fig. 5a, b.

The gate controllability is confirmed by measuring the dependence of the STM tip height on gate voltage (V$_g$). As V$_g$ increases from 0 to 20 V, the STM-tip retracts in a constant current mode indicating that charge has accumulated on the graphene surface (Fig. S2), reflecting successful gating. Similar to the V$_b$-dependent tip height, a large height change is observed in the relaxed case, indicating the polyimide film with less tension.

The change of surface morphology and its dependence on indentor height (V$_{st}$) also shows a similar behavior (Fig. S3). In the tented case, the obvious changes in the surface morphology is observed by increasing V$_{st}$. In contrast, in the relaxed case, the surface morphology is barely changed, and only a rigid shift of feature is observed with increasing V$_{st}$. Additionally, the height difference between the maximum and minimum in the tented case is 1.3–1.9 nm, which is lower than that of 2.8–3.4 nm in the relaxed case. It indicates that the tented polyimide film is more stretched and consequently flattened.

**Figure 6:**
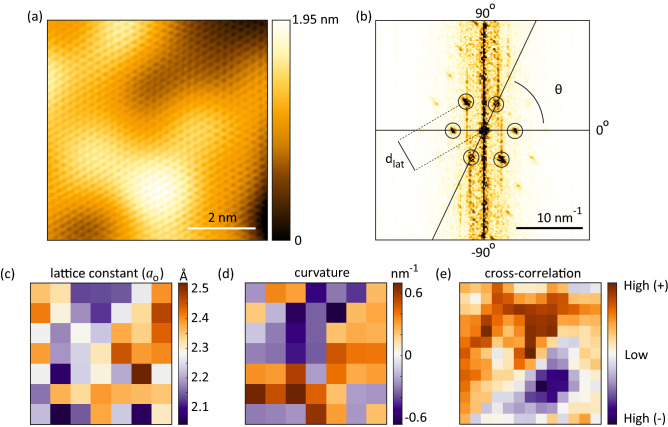
(**a**) Atomic-resolution image of graphene in the relaxed case and (**b**) its fast-Fourier transformation converted image with six clear lattice points. (**c**–**e**) The map of local average graphene lattice constant ($a_0$) and local curvature calculated from (**a**) and their cross-correlation.

The sample holder is stable enough for the topography measurement at the atomic level, which enables us to analyze the distribution of lattice parameters for calculating the strain. Figure 6a shows the atomic resolution STM image of the graphene/polyimide film in the relaxed case. The fast-Fourier transformation (FFT) converted image is shown in Fig. 6b. The lattice points in three directions can be clearly seen. The map of local lattice constants ($a_0$) averaged over three directions is shown in Fig. 6c. The median and standard deviation obtained by Gaussian-fitting the histogram of the lattice map are 2.27 Å and 0.11 Å, suggesting a range of strain of ±4.8% on graphene. The local curvature of substrate surface roughness has been reported to induce such fluctuation of $a_0$ by bending strain^4^. To verify this, we calculate the local curvature over the same small domains and plot its map in Fig. 6d. The calculated curvature map captures the feature of hill and valley of the surface morphology measured in Fig. 6a. Similarly, by calculating the cross-correlation coefficients, we found that the maps of local $a_0$ and curvature are highly positively correlated, indicated by a maximum value located at the center of Fig. 6e.

**Figure 7:**
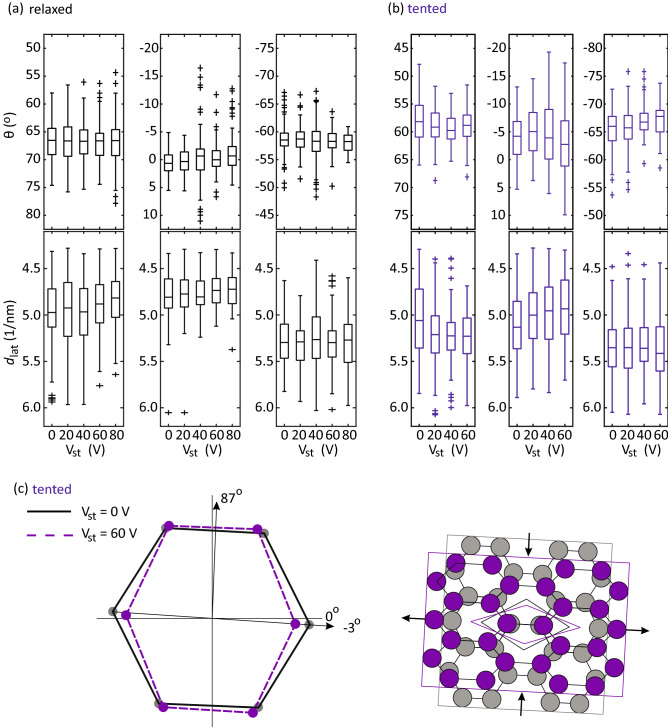
Characterization of strain-controllability. The boxplots of local graphene lattice points in (**a**) relaxed and (**b**) tented cases with increasing piezoelectric actuator voltage (V$_{st}$). For each lattice point, $d_{lat}$ is the length of reciprocal lattice vector, and $\theta$ is the angle defined by comparing with the horizon, 0$^\circ$-line drawn in (**c**). (**c**) The schematic summarises how graphene is deformed in the tented case when increasing V$_{st}$, based on the shifts in the medians extracted from (**b**). The changes in the schematic are exaggerated for clarity.

Figure 7a, b shows the boxplots of local graphene lattice points in both relaxed and tented cases with increasing V$_{st}$. For each lattice point, $d_{lat}$ is the length of the reciprocal lattice vector, and $\theta$ is the angle defined by comparing with the horizon, 0$^\circ$-line drawn in Fig. 7c. The scatter plots with marginal kernel densities are shown in Fig. S4. In the tented case, the median of $d_{lat}$-distribution in − 3$^\circ$ direction shifts from $\sim$ 5.13 to $\sim$ 4.93 nm$^{-1}$ when V$_{st}$ increases from 0 to 60 V, while its $\theta$-distribution remain unchanged. Moreover, the median of $d_{lat}$-distribution in $+$ 57$^\circ$ (− 66$^\circ$) direction shifts from $\sim$ 5.06 (5.34) nm$^{-1}$ to $\sim$ 5.22 (5.42) nm$^{-1}$, and the $\theta$-distribution in $+$ 57$^\circ$ (− 66$^\circ$) direction rotates counterclockwise (clockwise) with $\sim$ 2$^\circ$. On the other hand, in the relaxed case, except the median of $d_{lat}$-distribution in $+$ 67$^\circ$ direction and the median of $\theta$-distribution in $+$ 1$^\circ$ direction slightly shifts with increasing V$_{st}$, other distributions remain roughly the same. These overall shifts in distributions suggest that most of the areas in the tented case are affected by strain transferred from polyimide film, in contrast to the relaxed case where only a few local areas are changed, so no noticeable trend can be found.

To further verify the origin of the lattice parameter change, we measure STM images of graphene in the tented condition with three different V$_b$ of − 0.5 V, 0.5 V, and 1 V at a different area. We find that the results of graphene surface morphologies and lattice constant distributions remain largely unchanged with different V$_b$, as shown in Fig. S5. This indicates that the tip-sample force is sufficient to lift the sample height, as we have seen in Fig. 5d, but not enough to change the lattice parameters. Therefore, the change in $d_{lat}$ and $\theta$ described above is mainly caused by stretching due to increased indentation depth, not by the tip-induced curvature change during scanning. We present a schematic diagram in Fig. 7c to describe the changes in graphene reciprocal lattices and corresponding structure in real space in the tented case with increasing V$_{st}$. The reciprocal lattices are drawn based on the medians extracted from Fig. 7b and then are converted into the real space lattice. The changes in the schematic are exaggerated for clarity. It is shown that after increasing V$_{st}$ to 60 V, the graphene experiences an asymmetrical strain distribution, which stretches along $\sim -$ 3$^\circ$ (armchair) direction and compresses along $\sim$ 87$^\circ$ (zigzag) direction. The changes in the graphene’s reciprocal lattice, $G'=(I+\bar{\varepsilon })^{-1}G$, can be used to extract the strain tensor ($\bar{\varepsilon }$)^55^:

1 $$\begin{aligned} \bar{\varepsilon }=\begin{pmatrix} \varepsilon _A &{} \gamma _s\\ \gamma _s &{} \varepsilon _Z \end{pmatrix}, \end{aligned}$$

where $\gamma _s$ is the shear strain, $\varepsilon _A$ and $\varepsilon _Z$ are the uniaxial strain applied along armchair and zigzag direction, respectively. Based on the changes in the positions of medians in distributions when V$_{st}$ increases from 0 to 60 V, we found $\varepsilon _A\approx$4%, $\varepsilon _Z\approx$-4% and $\gamma _s\approx$0.

## Conclusions

We have developed a strain- and gate- controllable STM sample holder for 2D materials by employing an in-situ nanoindentation method. 2D materials supported by polyimide film with a thickness of $\sim$ 1 $\upmu$m are deformed by an indentor with a travel range of $\sim$ 120 $\upmu$m with nanometer precision using a gearbox in combination with a piezoelectric actuator. Since the sample holder is compact with a size of $\sim$ 160 mm$^2$ $\times$ 5.2 mm, it can be used not only in STM but also for many other analytic tools, such as AFM and Raman spectroscopy for comprehensive analysis. A series of atomic resolution STM images were obtained to examine the strain response of the graphene lattice to the indentation depth. The variation of STM tip height in the constant current mode was used to trace the polyimide film deflection in the indenting process. The change in the distribution of reciprocal lattices indicates the graphene in the measured area experience both a tensile strain of 4% in the armchair direction and a compressive strain of 4% in the zigzag direction, in addition to the bending strain induced by the polyimide surface roughness. We believe our direct observations of strain-induced structural change with atomic resolution provide valuable information for future strain engineering applications in 2D materials.

## Methods

### Sample preparation

The CVD-grown graphene and MoS$_2$ monolayer were purchased from 2D Semiconductors (CVD-Graphene-Cu and CVD-MoS2-ML-S). The polyimide film, with a thickness of $\sim$ 1 $\upmu$m, was fabricated on glass by using a spin-coating method (6000 rpm, Dupont, PI2610) from the Natural and Medical Sciences Institute at the University of Tübingen. To form the sample structure as shown in Fig. 2, we deposited a $\sim$ 20 nm Au film on the polyimide surface, detached the polyimide film by water intercalation, and subsequently turned the top and bottom upside down when transferring the polyimide film with a size of $\sim$ 3 $\times$ 3 mm$^2$ to the silicon substrate. 2D materials were transferred to the polyimide film supported by the silicon substrate using the wet etching method^39^. After the transfer of 2D material, a 30 nm Pd layer was deposited on the edge of 2D material through a circular-shaped shadow mask to enhance the strain transfer. Lastly, the polyimide film was transferred to the ceramic plate with the bottom fixed by the conductive epoxy (EPO-TEK, E4110). Au wires with a diameter of $\sim$ 17 $\upmu$m were used to connect both the Pd and Au contact to the sample holder electric pins. The metal deposition mentioned above were carried out in a metal depositor (Leybold, Univex 450) in the cleanroom with a flux of 1 Ås$^{-1}$ and base pressure $\le$ 1 $\times$ 10$^{-6}$ mbar.

### AFM

AFM (Bruker, Dimension Icon, OLYMPUS tip, OMCL-AC200TS-R3) in tapping mode was used to measure the macroscopic surface morphology of the bare polyimide film in air at room temperature. The images were analyzed using Gwyddion^56^.

### Raman spectroscopy

Raman spectroscopy (S&I GmbH) was carried out to characterize the macroscopic strain distribution of the monolayer MoS$_2$ transferred on the polyimide film in air at room temperature. A 488 nm laser focused by a 10$\times$ objective with a spot size of $\sim$ 10 $\upmu$m was used. To prevent thermal-induced artifacts, the incident laser power was kept < 0.12 mW. 1800 lines mm$^{-1}$ grating was used. The typical acquisition time was $\sim$5 mins.

### STM

The atomic-scale measurement on the graphene transferred on the polyimide film was performed in a home-built UHV STM at $\sim$ 4.8 K. The STM was operated in the constant current mode (100 pA, V$_b$ = 0.5 V). Given the temperature limitation of piezoelectric actuator (150 $^\circ$C) and the outgassing rate of polyimide, the graphene was kept heating below 100 $^\circ$C in UHV condition for two days for cleaning. Before introducing the sample holder to the STM cryostat, the indentor was positioned at the “contact” position as a reference. During the cooling process in the STM cryostat, the indentor position may drift due to the difference in thermal expansion coefficients of the various constituent materials of the strain sample holder. The gearbox rotation was done at room temperature to avoid any possible damage due to the high friction between the gear teeth at low temperature.

### Analysis of lattice parameters and local curvature

The lattice parameters and local curvatures were analyzed by using MATLAB with a home-built code. To calculate $d_{lat}$ and $\theta$ of local lattice points in each STM topography image, we divided the image into smaller domains with a size of $\sim$ 1 nm$^2$. Each area was then converted into reciprocal space by 2D fast Fourier transformation (FFT). Zero-padding was used to enlarge the converted image size, which improves the accuracy in the following fitting step. The Hann function was used as the window for FFT to enhance frequency resolution and reduce spectral leakage. A Gaussian blurring on the FFT-converted image was performed to reduce electronic noise for better identifying the lattice points. Bright spots in a smoothened image were considered, as lattice points when their intensities were above a certain threshold. To determine the position of lattice points, Gaussian fitting was used with five fitting parameters: the amplitude and two lateral widths and positions. Locations of lattice points were used to calculate $\theta$ and $d_{lat}$. The local maximum and minimum principal curvatures, $k_{max}$ and $k_{min}$, respectively, of the image were calculated by $k_{max}=H+\sqrt{(H^2-K)}$ and $k_{min}=H-\sqrt{(H^2-K)}$, where $H=\frac{1}{2}\text {tr}(S(p))$ is mean curvature and $K=\text {det}(S(p))$ is Gaussian curvature, and *S*(*p*) is shape operator at a point, *p*. The curvature-induced strain ($\varepsilon$) in Fig. 4a was calculated based on a pure bending model^4^: $\varepsilon =(d/2)/(1/R-d/2)\times 100$%, where *R* is the local radius of curvature and *d* is the thickness of the bent material. *R* was chosen to be $1/k_{max}$ or $1/k_{min}$, depending on the absolute magnitude in which one is smaller. *d* was chosen to be $\sim$ 1 $\upmu$m for polyimide film in Fig. 4a or $\sim$ 0.345 nm for graphene in Fig. 6d.

## Supplementary Information

Supplementary Information.

## Data Availability

The datasets used and/or analyzed during the current study available from the corresponding author on reasonable request.
