# Supplementary material for: In-situ atomic level observation of the strain response of graphene lattice
Source: Sci Rep. 2023 Feb 11;13:2451. doi: 10.1038/s41598-023-29128-4 (PMC9922254; doi:10.1038/s41598-023-29128-4)
Supplement: Supplementary file 1 — Supplementary Information. [file 41598_2023_29128_MOESM1_ESM.pdf]

Supporting information

In-situ atomic level observation of the strain response of graphene lattice

Jz-Yuan Juo[1], Bong Gyu Shin[1,2], Wolfgang Stiepany[1], Marko Memmler[1], Klaus Kern[1,3], and Soon Jung Jung[1,*]
[1]Max-Planck-Institut für Festkörperforschung, Heisenbergstraße 1, DE-70569 Stuttgart, Germany
[2]SKKU Advanced Institute of Nanotechnology (SAINT), Sungkyunkwan University (SKKU), Suwon 440-746, Republic of Korea
[3]Institut de Physique, École Poly-technique Fédérale de Lausanne, CH-1015 Lausanne, Switzerland
*corresponding author

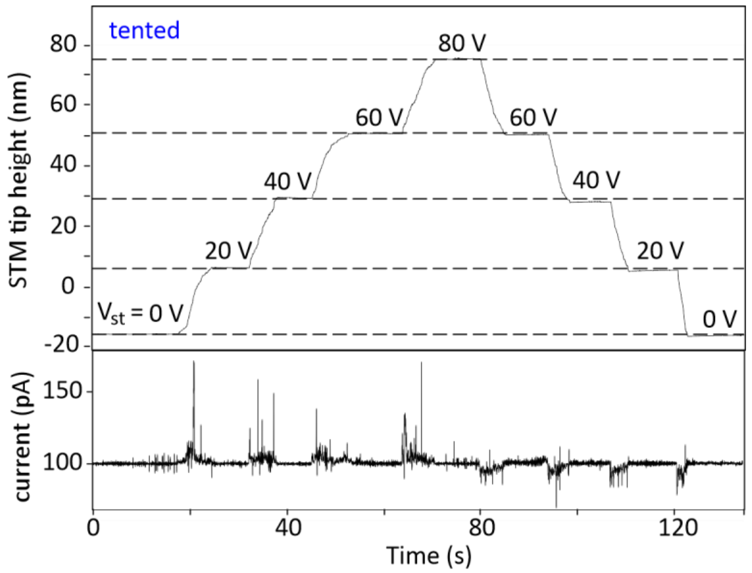

FIG. S1. Reversible graphene/polyimide height changes by increasing the voltage applied to the piezoelectric actuator carrying the indentor (V_{st}) in the tented condition. STM in a constant current mode (0.5 V, 100 pA).

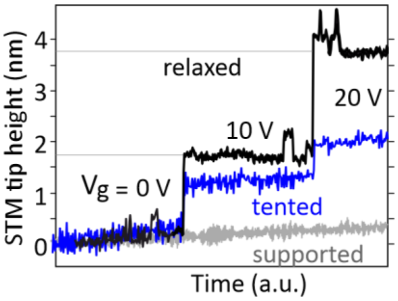

FIG. S2. Characterization of gate-controllability. STM-tip height feedbacks how the sample height is changed by increasing gate voltage (V_g).

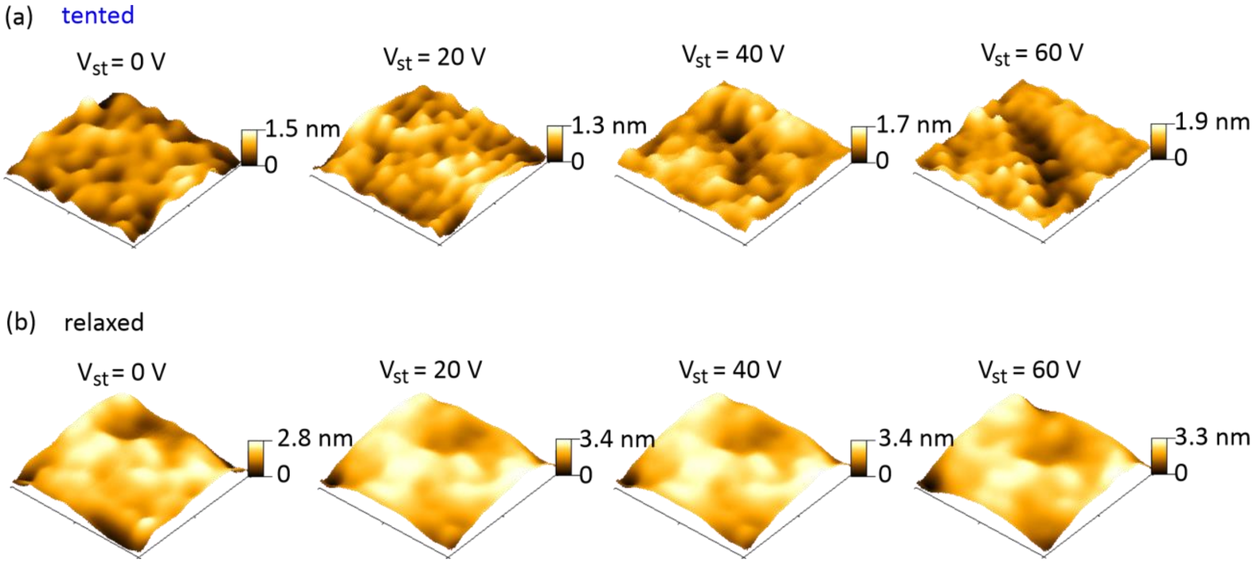

FIG. S3. The measured STM topography images with increasing indentor height (V_{st}). (a) tented and (b) relaxed cases. The size of images is 15 nm x 15 nm.

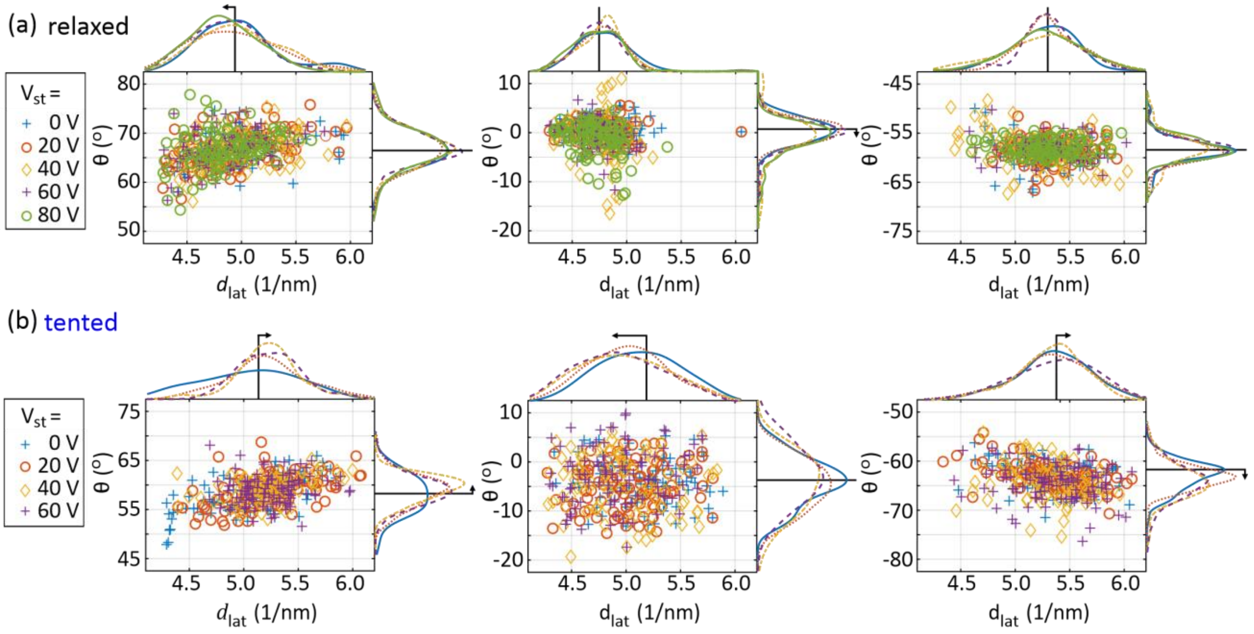

FIG. S4. The scatter plots with marginal kernel densities of local graphene lattice points in (a) relaxed and (b) tented case with increasing the piezoelectric actuator voltage (V_{st}). For each lattice point, d_{lat} is the length of reciprocal lattice vector, and θ is the angle defined by comparing with the horizon, 0°-line drawn in Fig. 8(c).

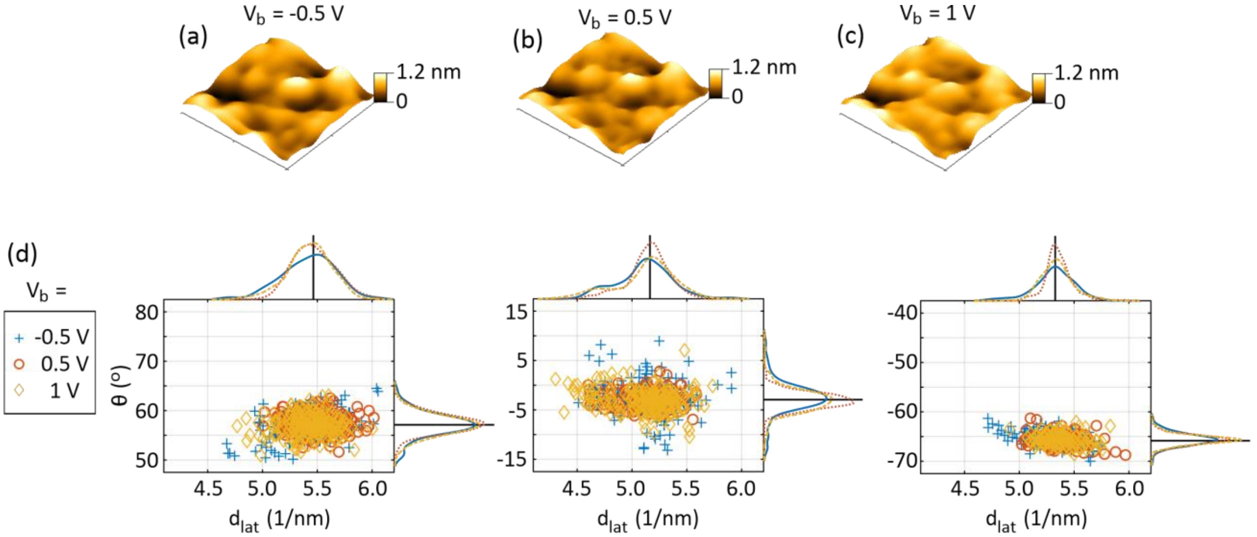

FIG. S5. (a-c) STM topography images measured at the same area in the tented case with different bias voltages (V_b). (d) The scatter plots with marginal kernel densities of local graphene lattice points analyzed from (a-c). The size of images is 15 nm x 15 nm.
